# Hidden Cardiovascular Anatomy in “Saint John the Baptist” by Leonardo da Vinci

**DOI:** 10.1055/s-0042-1742698

**Published:** 2022-08-07

**Authors:** Grigol Keshelava

**Affiliations:** 1 Department of Vascular Surgery, Clinic “Helsicore”, Tbilisi, Georgia; 2 Department of Vascular Surgery, New Vision University, Tbilisi, Georgia

**Keywords:** cardiovascular anatomy, Saint John the Baptist, Leonardo da Vinci

## Abstract

Leonardo da Vinci conducted many anatomical studies during his life. Today, almost the complete set of these anatomical drawings and comments is owned by the British Crown and resides in the Royal Library at Windsor Castle, United Kingdom.

Through the program Paint X, we moved two details on the painting “Saint John the Baptist.” The moving details are circled along the faint contour by Leonardo da Vinci himself. We obtained heart and aortic arch imaging.

## Introduction

“Saint John the Baptist” is a High Renaissance oil painting on walnut wood by Leonardo da Vinci. It is believed to be his final painting. The original size of this work was 69-by-57 cm. St John emerges from a dark background his skin and hair ringlets are lit in what is a fine example of Leonardo's sfumato.

## Leonardo da Vinci's Anatomy of the Heart and Vessels

Leonardo da Vinci (1452–1519) conducted many anatomical studies during the second half of his life and his drawings not only demonstrate his artistic genius but also prove that he was a great scientist. Today, almost the complete set of these anatomical drawings and comments is owned by the British Crown and resides in the Royal Library at Windsor Castle, United Kingdom. 1 Leonardo's early anatomical studies were not systematic in nature. Rather, he sought to study not only the anatomy of the body but also to explain the relationship between the structure of the body in all its aspects and the conception, growth, and the expression of the emotions. It was not until later in his life that he embarked on a fundamental study of anatomy. 2

The vast majority of Leonardo's studies on the heart are reported in drawings and notes he produced in Milan from 1508 to 1513, though afterwards he continued to work in Rome. 3 He made several wax casts of the bull heart, and from these casts, he constructed glass models to study the hydraulic characteristics of blood flow through the heart and its valves. Seeds were used to visualize turbulences and blood flow. He also studied the dynamics of water flow in rivers, using colors to show the flow patterns. Leonardo translated those findings to blood flow in vessels. 4 5

The master studied the anatomy of blood vessels in detail and concluded that the aorta nourishes all the body. He suggested that the bronchial arteries receive freshness from the bronchi and that venous blood receives freshness in the lungs before returning to the heart. By examining the coronary arteries, he came to the conclusion that the heart feeds itself. 5 6

Due to his knowledge of hydrodynamics and anatomy, Leonardo was able to interpret the physical basis of such a natural death, such as the atherosclerosis of old age. 6 7

He believed that the heart consisted of four chambers, and he made a functional distinction between atria and ventricles. 8 Unaware of the connection of circulatory system that would not be fully demonstrated until 1628 by William Harvey, Leonardo's ideas followed Galenic percepts: the blood was produced in the liver, cooled by the lungs, and pumped by the heart. 9

In this period, anatomical knowledge in Europe was largely based on manuscripts from classical Greece and medieval Italy, the dissection of animals, and the occasional autopsy dissection of a condemned criminal. 10 11 Despite the fact that cadaver dissection was illegal in this epoch, physicians still managed to deepen knowledge in human anatomy. Leonardo fundamentally studied the anatomy and used this knowledge in his art. Leonardo filled notebooks with carefully drawn two-dimensional representations of the organs, tissues, and skeletal formations uncovered during his dissections. 12

Hidden anatomical elements in his paintings have also been uncovered by other artists of the Renaissance era. Researchers have found that the pomegranate in Botticelli's “Madonna of the Pomegranate'' corresponds to the appearance of the heart. 13 Also noteworthy is the “Creation of Adam” by Michelangelo. Researchers concluded that this work describes the anatomy of the human brain. 14

## Painting, “Saint John the Baptist'' by Leonardo da Vinci

**Fig. 1:**
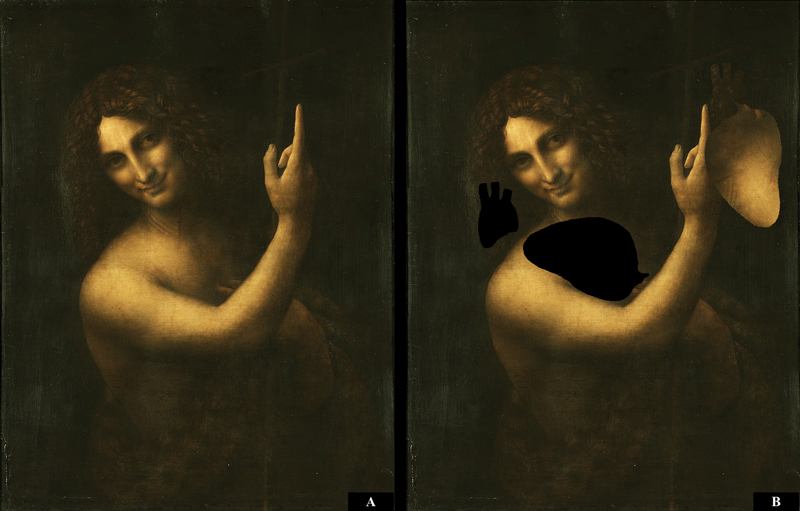
( **A** ) “Saint John the Baptist'' by Leonardo da Vinci (1513-1516). ( **B** ) The image obtained after moving the detail.

Traditionally, this painting has been considered the artist's last, dated to 1513 to 1516 ( Fig. 1A ). It is believed that the sfumato technique in this painting reaches the highest level. 15 The painting depicts Saint John the Baptist dressed in leather with a mysterious smile on his face. His left hand holds the cross, while his index finger of the right hand points toward the sky.

Following the French Revolution, this painting entered the collection at the Louvre, where it remains to this day.

Restoration at the Center for Research and Conservation for France's Museums (headed by researchers) commenced in October 2015. An initial radiological diagnostic attributed the discoloration to 17 layers of extremely oxidized varnish, 110 µm thick. Detail was revealed by removing 15 layers of varnish. The researchers encountered and removed several areas of ancient poorly executed overpainting, particularly in the Saint's arms and torso. X-rays also revealed that Leonardo repeatedly retouched the painting until the end of his life in 1519. The painting was put back on display in November 2016.

There is opinion that Leonardo already suffered from right hemiparesis while working on this painting and it is possible that the master indicated with raised Saint John's right arm the Paradise, where he will be young again and completely healthy. 16

## Interpretation of the painting, “Saint John the Baptist'' by Leonardo da Vinci

The object of this research is a “Saint John the Baptist'' by Leonardo da Vinci before restoration ( Fig. 1A ).

**Fig. 2:**
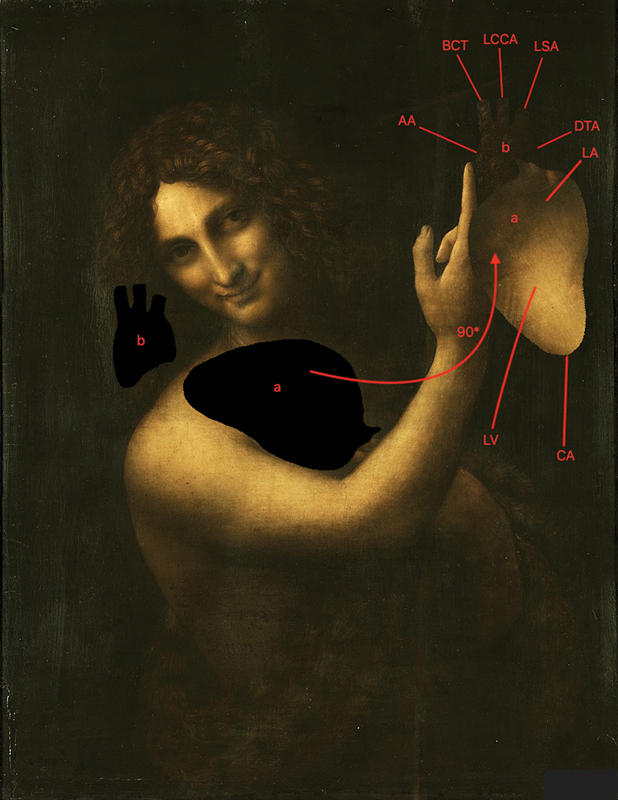
( **a** , **b** ) Initial and final position of moving details. The red arrow indicates the direction and angle of rotation of the detail ( **a** ). LA, left atrium; LV, left ventricle; CA, cardiac apex; AA, ascending aorta; BCT, brachiocephalic trunk; LCCA, left common carotid artery; LSA, left subclavian artery; DTA, descending thoracic aorta.

Through the program Paint X, we moved two details (a, b) ( Fig. 2 ) to the final locations. In addition, we rotated the detail (a) 90 degrees counterclockwise. The moving detail is circled along the faint contour by Leonardo da Vinci himself. The received heart imaging has an exact anatomical location and inclination. It reflects the left atrium, left ventricle, cardiac apex, ascending aorta, brachiocephalic trunk, left common carotid artery, left subclavian artery, and descending thoracic aorta ( Fig. 2 ).

The harmonious combination of the colors of the heart and aortic arch with the colors of neighboring details is remarkable ( Fig. 1B ).

As for the displacement of details in the painting, we see that in some of Leonardo's works the content changes as the details move. He sometimes hides things in his art work. In a similar way, we found the respiratory system in the, “Portrait of a Musician” and the anatomy of the heart and aortic arch in the painting, “Dreyfus Madonna.” 17 18

Why did Leonardo decide to describe the heart and blood vessels in association with St John the Baptist? We think we should look for the answer in the Bible, where it is written:

“God predestined John the Baptist to preach a gospel of baptism of repentance for the forgiveness of sins that prepared the hearts of men for the Lord Jesus Christ's coming” (Luke 3:3-4). The master symbolically connects the human hearts mentioned in the Bible with cardiovascular anatomy.

## Conclusion

In our opinion, Leonardo da Vinci secretly reflected the human heart with aortic arch in, “Saint John the Baptist.” An image obtained by moving two details of this painting shows the left atrium, left ventricle, cardiac apex, ascending aorta, brachiocephalic trunk, left common carotid artery, left subclavian artery, and descending thoracic aorta.
